# Machine learning-featured Secretogranin V is a circulating diagnostic biomarker for pancreatic adenocarcinomas associated with adipopenia

**DOI:** 10.3389/fonc.2022.942774

**Published:** 2022-08-17

**Authors:** Yunju Jo, Min-Kyung Yeo, Tam Dao, Jeongho Kwon, Hyon‐Seung Yi, Dongryeol Ryu

**Affiliations:** ^1^ Department of Molecular Cell Biology, Sungkyunkwan University (SKKU) School of Medicine, Suwon, South Korea; ^2^ Department of Pathology, Chungnam National University School of Medicine, Daejeon, South Korea; ^3^ Department of Medical Science, Chungnam National University School of Medicine, Daejeon, South Korea; ^4^ Laboratory of Endocrinology and Immune System, Chungnam National University School of Medicine, Daejeon, South Korea

**Keywords:** pancreatic cancer, pancreatic adenocarcinoma, biomarker, diagnosis, prognosis, machine learning, adipopenia, cachexia

## Abstract

**Background:**

Pancreatic cancer is one of the most fatal malignancies of the gastrointestinal cancer, with a challenging early diagnosis due to lack of distinctive symptoms and specific biomarkers. The exact etiology of pancreatic cancer is unknown, making the development of reliable biomarkers difficult. The accumulation of patient-derived omics data along with technological advances in artificial intelligence is giving way to a new era in the discovery of suitable biomarkers.

**Methods:**

We performed machine learning (ML)-based modeling using four independent transcriptomic datasets, including GSE16515, GSE62165, GSE71729, and the pancreatic adenocarcinoma (PAC) dataset of the Cancer Genome Atlas. To find candidates for circulating biomarkers, we exported expression profiles of 1,703 genes encoding secretory proteins. Integrating three transcriptomic datasets into either a training or test set, ML-based modeling distinguishing PAC from normal was carried out. Another ML-model classifying long-lived and short-lived patients with PAC was also built to select prognosis-associated features. Finally, circulating level of SCG5 in the plasma was determined from the independent cohort (non-tumor = 25 and pancreatic cancer = 25). We also investigated the impact of SCG5 on adipocyte biology using recombinant protein.

**Results:**

Three distinctive ML-classifiers selected 29-, 64- and 18-featured genes, recognizing the only common gene, *SCG5*. As per the prediction of ML-models, the *SCG5* transcripts was significantly reduced in PAC and decreased further with the progression of the tumor, indicating its potential as a diagnostic as well as prognostic marker for PAC. External validation of SCG5 using plasma samples from patients with PAC confirmed that SCG5 was reduced significantly in patients with PAC when compared to controls. Interestingly, plasma SCG5 levels were correlated with the body mass index and age of donors, implying pancreas-originated SCG5 could regulate energy metabolism systemically. Additionally, analyses using publicly available Genotype-Tissue Expression datasets, including adipose tissue histology and pancreatic *SCG5* expression, further validated the association between pancreatic *SCG5* expression and the size of subcutaneous adipocytes in humans. However, we could not observe any definite effect of rSCG5 on the cultured adipocyte, in 2D *in vitro* culture.

**Conclusion:**

Circulating SCG5, which may be associated with adipopenia, is a promising diagnostic biomarker for PAC.

## Introduction

With a nine percent five-year survival rate, pancreatic adenocarcinoma (PAC) is the 7th deadly cancer (1, 2). Its early detection is difficult due to the absence of distinguishing symptoms and particular biomarkers. The development of distinct biomarkers is hindered because the specific cause of pancreatic cancer is unclear, but this is extremely necessary to facilitate early-stage diagnosis and therapies.

Several serum biomarkers for PAC are widely used in clinical practice, including carbohydrate antigen 19-9 (CA19-9), CA242, and carcinoembryonic antigen (3). CA19-9 is an isolated version of the sialylated Lewis antigen that is commonly used in clinical settings to diagnose pancreatic cancer (4). However, in addition to malignant cells, it is widely produced and shed by both normal and benign pancreatic cells. Moreover, patients with other gastrointestinal malignancies such as gastric, biliary, or colon cancer can have increased CA19-9 levels (5). CA242, another serum biomarker for the diagnosis of PAC, is also elevated in patients with other malignant tumors, such as cervical, colon, esophageal, and ovarian cancers as well as lymphoma (6–8). Patients with other malignancies, such as colorectal and non-small cell lung cancer, have also been documented to have elevated levels of serum carcinoembryonic antigen, another blood biomarker for PAC (7, 9). Moreover, it was also elevated in heavy smokers (10). Thus, a serum biomarker unique to pancreatic cancer is urgently required.

Cachexia, which includes involuntary anorexia, inflammation, insulin resistance, hypogonadism, and anemia leading to sarcopenia and adipopenia, is a dangerous consequence that occurs frequently in patients with advanced chronic diseases such as chronic kidney disease, chronic obstructive pulmonary disease, diabetes, cancer, and congestive heart failure (11–13). Cancer cachexia (CC) is a multifactorial syndrome defined by the gradual wasting of muscle and fat mass that cannot be entirely reversed by traditional dietary assistance (14). It is known that its rate of incidence is high in pancreatic, gastric, lung, and colon cancers (12, 13). A mixture of systemic factors, including endocrine factors, cytokines, and metabolites, originating from cancer cells and the tumor microenvironment can trigger of CC, altering metabolism and causing mitochondrial dysfunction, browning of the white adipose tissue, anabolic resistance and catabolic subservience, and central nervous system disturbances (15–19). In CC, extensive loss of skeletal muscle and adipose tissue is a severe syndrome linked with significant morbidity and mortality (20–22). Around 80% of pancreatic cancer patients have CC at some point during their illness (23, 24). Some traits of CC are associated with poor prognosis (13), implying that any prognostic marker may mediate of CC or couple with it.

One of the finest methods to deduce high-potential candidates is machine-learning-based categorization, which acts as a researcher’s bias-free technique to discover traits that may function as diagnostic and prognostic biomarkers, giving an opportunity to uncover underlying pathophysiological principles and mechanisms (25–28). Herein, we performed machine learning-based classification to identify diagnostic and prognostic markers for PAC. To mine a biomarker having predictive potential for both diagnosis and prognosis of PAC, we focused on finding the common features of machine learning-based classification using the transcriptomes from normal human pancreas (NOP) and PAC of three independent cohorts as well as the PAC transcriptomes from The Cancer Genome Atlas (TCGA). Then, using data from an independent cohort, we confirmed that plasma levels of the discovered common feature (Secretogranin V (SCG5)) was higher in patients with PAC than in patients without it. Finally, investigations employing publicly accessible Genotype-Tissue Expression (GTEx) datasets, such as adipose tissue histology and transcriptome, confirmed the association between pancreatic *SCG5* expression and white adipose tissue size in patients with PAC, implying that SCG5 could be a humoral factor mediating CC.

## Materials and methods

### Human study

From January 2011 to December 2019, the Biobank of Chungnam National University Hospital, a Korea Biobank Network, provided 50 plasma samples from PAC and normal (no PAC, control) patients. All patients’ pre-operative blood samples (taken within one week before the operation) were collected, processed to extract plasma, and kept in liquid nitrogen. PAC patients included 13 men and 12 women, with ages between 48 to 86 years, varying range of body mass index (BMI) (16.68-26.9), and progression of cancer between stages IIB to IV.

### Data collection and processing pancreatic transcriptomes for machine learning modeling

From the Gene Expression Omnibus (GEO) of the National Center for Biotechnology Information (NCBI), we used a publicly accessible transcriptome dataset with accession numbers GSE16515, GSE62165, and GSE71729, as well as from TCGA PAC (also known as PAAD). Three studies included 52, 131, and 191 donors, with 16, 13, and 46 being healthy and 36, 118, and 145 being patients with PAC, respectively. The TCGA PAC study included 177 subjects, with 21 being stage I, 146 being stage II, and seven being stage III+IV. Secretory genes were made up of 1,703 genes that could be detected in the blood and serve as biomarkers for the diagnosis and prognosis of PAC (29). As indicated in the Human Protein Atlas (30), we defined the list of 1,703 genes encoding secretory proteins (hereafter, secretory genes, downloaded on 2020.11.11, https://www.proteinatlas.org).

### Construction and evaluation of machine learning models

Construction of models with Random Forest (RF) algorithm was done as described previously (28). To build models capable of distinguishing between NOP and PAC samples as well as higher and lower survival rates, we implemented RF algorithm-based machine learning with optimized parameters through a 10-fold repeated cross-validation using the R package “*caret*”. The model was constructed by gradually reducing the number of variables for discriminating between NOP and PAC samples to 29, 10, 8, 6, 4, and 2. The model that differentiates between greater and lower survival rates was evolved by gradually lowering the number of features to 1,703, 1,024, 512, 256, 128, 64, 32, 16, 8, and 4. The GINI importance value was used to describe the importance of features gained from each model developed in this manner. We also applied the XGBoost algorithm with optimized parameters through 5-fold cross-validation to construct models that distinguish between higher and lower survival rates. The built-in function of the R package “*xgboost*” was used to determine the key features of models. We computed the area under the receiver operating characteristics (AUROC), accuracy, kappa, and F1-score using R package “*MLmetrics*” to assess the performance of the constructed ML models.

### Plasma SCG5 quantification using ELISA

In order to gauge plasma SCG5 levels, human SCG5 enzyme-linked immunosorbent assay (ELISA) kits were used (SCG5 ELISA Kit, MBS2883472, MyBioSource Inc., San Diego, CA, USA). All ELISAs were conducted at the same time, as recommended by the manufacturer. Briefly, the sandwich ELISA was carried out as follows: A human SCG5-specific antibody was pre-coated on the microtiter plate supplied. In duplicates, standard, blank, and samples (100 µl each) were introduced to the wells. Plates were sealed with a plate sealer and incubated at 37°C for two hours. Each well’s liquid was withdrawn without being washed, and each well received 100 µl of the working solution of Detection Reagent A (biotin-labeled antibody) diluted at 1:100 with the assay Diluent A, followed by an hour of incubation at 37°C. The culture dishes were then incubated for an hour after being five times rinsed in wash buffer at 37°C with 100 µl per well of the working solution of Detection Reagent B (avidin–HRP, horseradish peroxidase conjugates) diluted at 1:100 with the Assay Diluent B. After washing five times with wash buffer, specific binding was detected for 20 min at 37°C with 90 µl of substrate solution (TMB, HRP Substrate). After stopping the reactions with 50 µl of the stop solution, the plates were read immediately at 450 nm using an automated microplate reader (SpectraMax 190, Molecular Devices LLC, San Jose, CA, USA). SoftMax Pro 4.8 software was used to calculate SCG5 concentrations by taking the mean OD of each standard and sample.

### Assessment of the cross-sectional area of human adipose tissue

Hematoxylin and eosin-stained histology images of subcutaneous adipose tissue (SAT) for cross sectional area (CSA) estimation were accessed by sample ID at the GTEx portal (https://gtexportal.org/). The measurement of the CSA of adipocytes was carried out with the open-source image analysis software Fiji, also known as ImageJ (https://imagej.net/software/fiji/downloads). The plugin of the *Cross-Sectional Analyzer* was downloaded and set up for image analysis according to manual instructions (https://imagej.net/plugins/cross-sectional-analyzer). Image preprocessing includes converting images from SVS format to TIFF files at 10x magnification of random tissue locations, then importing the images into Fiji. The monochromatic images were created from TIFF files and put to a green channel for subsequent processes. CSA was automatically calculated by the *Cross-Sectional Analyzer* after manual adjustment for any misrecognition of the cell membrane. Finally, using the aforesaid analysis results, we compared the CSA of the two sample groups (the high- and low-expressed *SCG5*). The student’s t-test was performed as a statistical test to deduce the difference in mean between the two groups. The frequency of each CSA interval is calculated by dividing the percentage of cells with a CSA in that range by the total number of cells in each sample.

### Gene set enrichment analysis

GSEA was carried out as previously reported (31). The GSEA Java desktop program (GSEA v. 4.1.0 for Windows) was downloaded from the website of Broad Institute (https://www.gsea-msigdb.org).

### Adipocyte culture, treatment of recombinant SCG5 protein, oil-red-o-staining and real-time quantitative reverse transcription polymerase chain reaction

The base medium, Dulbecco’s modified Eagle’s media with 25 mM glucose, 10% fetal bovine serum, 100 µg/ml streptomycin, and 100 U/ml penicillin, was used to grow 3T3L-1 cells under the guidance of the American Type Culture Collection (www.atcc.org). This was done at 37°C in a humid environment with 5% CO2. To differentiate adipocytes, 3T3L-1 cells were maintained for an additional two days with 100% confluence (day 0), then replaced and maintained for two days with differentiation medium (1.0 µg/ml insulin, 1 µM dexamethasone, and 0.5 mM 1-methyl-3-isobutylxanthine), and then kept in basal medium supplemented with 1.0 µg/ml insulin alone. The cells were fed with baseline media supplemented with or without recombinant SCG5 protein diluted in PBS on the 4th day of differentiation. Recombinant SCG5 protein was treated at concentrations of 50, 100, or 200 ng/ml, which is determined by the plasma concentration of SCG5 in human donors. The medium was replaced every second day. Then the lipid accumulation was evaluated.

As previously mentioned, neutral lipids were stained with Oil-Red-O (32). In brief, to quantify intracellular lipid accumulation, 3T3-L1 preadipocytes were differentiated into adipocytes in 12-well plates and treated with or without recombinant SCG5 on day 4 of differentiation for 4 days (day 8). The procedure for staining cells included washing them twice with PBS, fixing them in 4% formaldehyde for 15 minutes, staining them for 20 minutes at room temperature with 0.5% Oil-Red-O in 60% isopropanol, and then washing them five times with distilled water. Oil-Red-O-stained lipid droplets were photographed and photographed under a light microscope before being extracted with 100% isopropanol for absorbance (490 nm wavelength) measurement with a SpectraMax Plus 384 microplate spectrophotometer (Molecular Device, CA, USA).

Semi-quantitative real-time polymerase chain reaction (qRT-PCR) was carried out as previously reported (31). Using the RNeasy Mini Kit (Qiagen, Netherlands) to extract total RNA from the cells treated with or without rSCG5, cDNA was produced using the PrimeScript™ RT reagent kit (TAKARA, Japan) from total RNA (1 µg) in accordance with the manufacturer’s instructions. SYBR Green I was used to conduct qRT-PCR with diluted cDNA in an end volume of 10 µl reaction mixtures (QuantStudio 6 Flex Real Time System, Applied Biosystems, MA, USA). The relative differences in mRNA expression levels were determined according to the comparative Ct methods that were normalized to *Mrpl32* mRNA. Primer sequences are summarized in **Supplementary Table 1**.

### Statistical analysis and visualization

The Shapiro–Wilk test was utilized to determine the normality of data distribution. To examine the difference in mean values between two or more groups, the student’s t-test, Wilcoxon rank-sum test, one-way analysis of variance, Kruskal-Wallis test, and Tukey’s Honest Significant Difference test were used, depending on the distribution. The threshold for statistical significance was a p value of 0.05 or lower. For survival analysis evaluating prognosis, we first divided the samples into two groups (*SCG5* high and low) by constructing ROC curves with the R package *multipleROC*, which determined optimal grouping with respect to the *SCG5* expression level. Then, probability of survival was evaluated with Kaplan–Meier curves. R Studio (ver. 2021.09.1 Build 372, https://www.rstudio.com/), R (ver. 4.1.2, https://www.r-project.org/), and the R packages *“ggpubr”, “dplyr”, “stringr”, “DescTools”, “multipleROC”, “reshape2”, “survminer”, “survival”, “RColorBrewer”, “pROC”, “igraph”, “ggraph”, “corrr”, “corrplot”, “ggplot2”*, and *“ggarrange”* were used for statistical analysis and visualization.

## Results

### Machine learning modeling to identify diagnostic and prognostic markers for pancreatic adenocarcinoma

**Figure 1:**
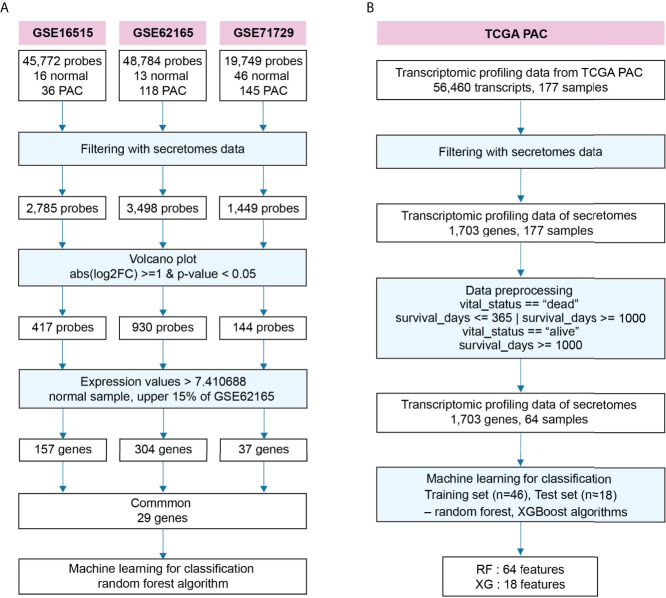
Schematic workflow to identify diagnostic and prognostic markers for pancreatic adenocarcinoma. **(A)** Workflow to identify prospective diagnostic markers from three independent transcriptomes. **(B)** Workflow to predict a putative prognostic marker from the transcriptome of pancreatic adenocarcinoma (PAC) at The Cancer Genome Atlas (TCGA).

To find blood-circulating diagnostic markers that can distinguish between NOP and PAC, we downloaded three transcriptome datasets, GSE16515, GSE62165, and GSE71729, from the Gene Expression Omnibus of the National Center for Biotechnology (**Figure 1A**) (33–35). From this, we filtered 1,703 secretory genes as stated in the ‘materials and methods’ section (30), after which we obtained 2,785 probes for GSE16515, 3,498 probes for GSE62165, and 1,449 probes for GSE71729, including duplicates. To further narrow down the candidates for blood diagnostic markers, differential gene expression analysis was conducted with the following cut-off criteria: only those genes whose absolute log2 fold-change was altered more than one-fold with statistical significance were selected between the two groups (NOP vs PAC). As a result, 417, 930, and 144 probes were obtained from each data set, respectively. Since the goal of the experiment was to identify prospective candidates for blood biomarkers that can be used in clinical practice, we employed additional cut-off criteria based on gene expression signal. Only genes corresponding to the top 15% of normal tissue expression signals (i.e., > 7.410688) were chosen. Each transcriptome data set qualified 157, 304, and 37 secretory genes from each respective data set for the next round. We selected the probe with the strongest signal if there were more than two probes for a gene. Finally, 29 common genes were identified from three independent pancreatic transcriptomes and applied to the RF algorithm-based supervised machine learning modeling.

We also built another RF algorithm-based model for detecting prognostic markers in blood, categorizing short- and long-lived candidates (**Figure 1B**). First, we downloaded data from TCGA which included data on survival days as well as transcriptomic data. Similar to finding diagnostic markers, we filtered for secretory genes and found 1,703 genes. The survival days in the data were ambiguous when the sample’s vital status was indicated as alive because it may have contained missing data recognized as alive. Thus, the data was classified as either short or long based on the following two criteria: first, we labeled survival days as “short” or “long” when the samples were dead, and only “long” when they were alive. Second, the term “short” was used if the survival days were less than 365, and the term “long” was used if they were greater than 1000. As a result, 64 samples were selected from a total of 177 samples. Then it was applied to the supervised RF- and XGBoost-algorithms, which were optimized by sequentially reducing the number of features as summarized in **Figure 1B**.

### ML-modeling using RF and XGBoost proposed SCG5 as a diagnostic and prognostic marker for pancreatic adenocarcinoma

**Figure 2:**
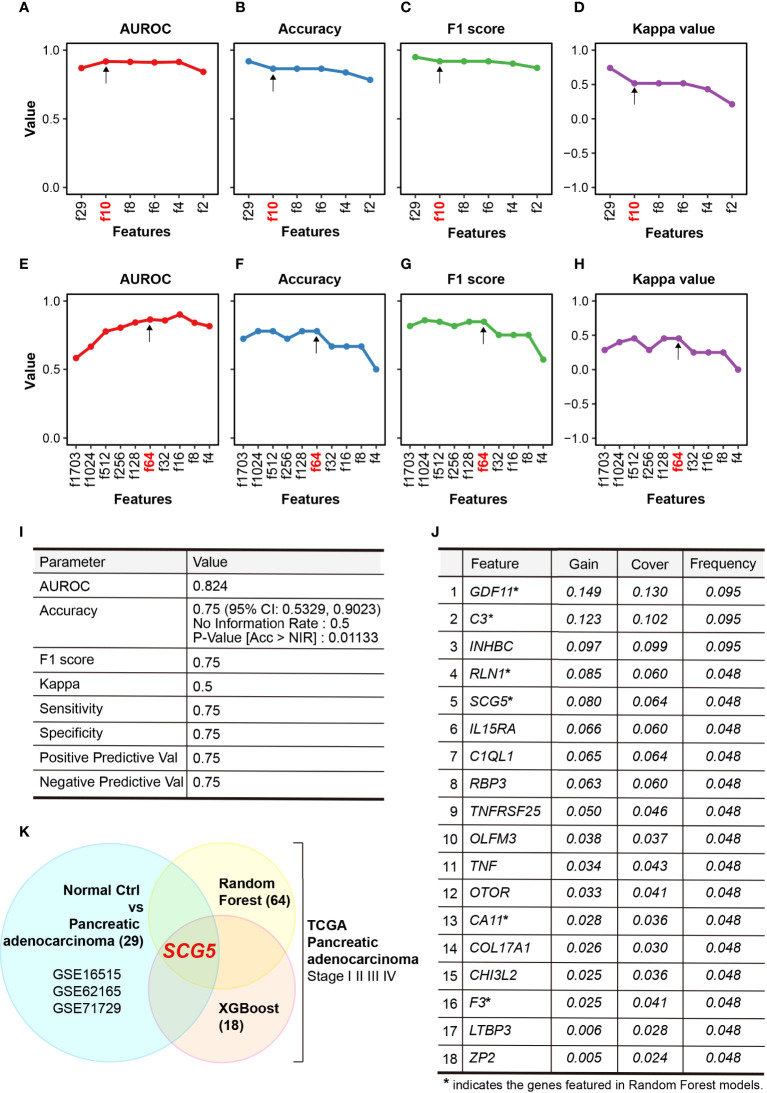
Performance of constructed random-forest (RF) models. **(A–D)** Plots summarizing the area under receiver operating characteristic curve (AUROC) **(A)**, accuracy **(B)**, F1 score **(C)**, and kappa value **(D)** of RF models that classifies normal pancreas and pancreatic adenocarcinoma. **(E, F)** Plots highlighting the AUROC **(E)**, accuracy **(F)**, F1 score **(G)**, and kappa value **(H)** of RF models that characterize the prognosis of PAC. **(I, J)** Tables summarizing the performance **(I)** and selected features **(J)** of XGBoost modeling, **(K)** Venn diagram showing *SCG5* as the only common feature in two distinct ML models.

The AUROC in the train set and accuracy, F1 score, and kappa value in the test set were calculated to evaluate the performance of each developed RF model to predict diagnostic markers (**Figures 2A–D**). In the order of features f29, f10, f8, f6, f4, and f2, each AUROC is 0.870, 0.981, 0.915, 0.911, 0.914, or 0.842, respectively. Each accuracy is 0.919, 0.865, 0.865, 0.838, or 0.784, each F1 score is 0.950, 0.919, 0.919, 0.902, or 0.871, and each kappa value is 0.741, 0.517, 0.517, 0.431, or 0.212, respectively. The model with 29 features (f29) had the highest accuracy, F1 score, and kappa values, but had a low AUROC. While it has a smaller number of features compared with f29, the model with 10 features (f10) had a high AUROC, and its accuracy and F1 score were comparable to those with 29 (f29). Therefore, we chose f10 as the optimal model. Evaluation of the prognostic markers by RF-algorithm was carried out in the same manner as the diagnostic markers (**Figures 2E–H**). AUROC is 0.582, 0.665, 0.775, 0.803, 0.841, 0.862, 0.856, 0.899, 0.839, and 0.814, and accuracy is 0.722, 0.778, 0.778, 0.722, 0.778, 0.778, 0.667, 0.667, 0.667, and 0.500, while the F1 score is 0.815, 0.857, 0.846, 0.815, 0.846, 0.846, 0.750, 0.750, 0.750, and 0.571, and kappa value is 0.286, 0.400, 0.455, 0.286, 0.455, 0.455, 0.250, 0.250, 0.250, and 0.00 for the respective features. We chose a model with 64 features (f64) to find the best model for classifying survival days into short and long. Evaluation of the prognostic markers by XGBoost-algorithm was also carried out in the same manner as previously described (**Figure 2I**). The AUROC is 0.824, the accuracy is 0.75 (95% CI: 0.5329, 0.9023), the F1 score is 0.75, and the kappa value is 0.5. The XGBoost-selected important features are *GDF11, C3, INHBC, RLN1, SCG5, IL15RA, C1QL1, RBP3, TNFRSF25, OLFM3, TNF, OTOR, CA11, COL17A1, CHI3L2, F3, LTBP3*, and *ZP2*. (**Figure 2J**). We found common genes from three independent ML-models that could differentiate between NOP and PAC as diagnostic markers and distinguish survival days based on medical history as prognostic markers. Finally, *SCG5* was obtained as a potential biomarker for PAC (**Figure 2K**).

### *SCG5* expression patterns and clinicopathological characteristics in NOP and PAC

**Figure 3:**
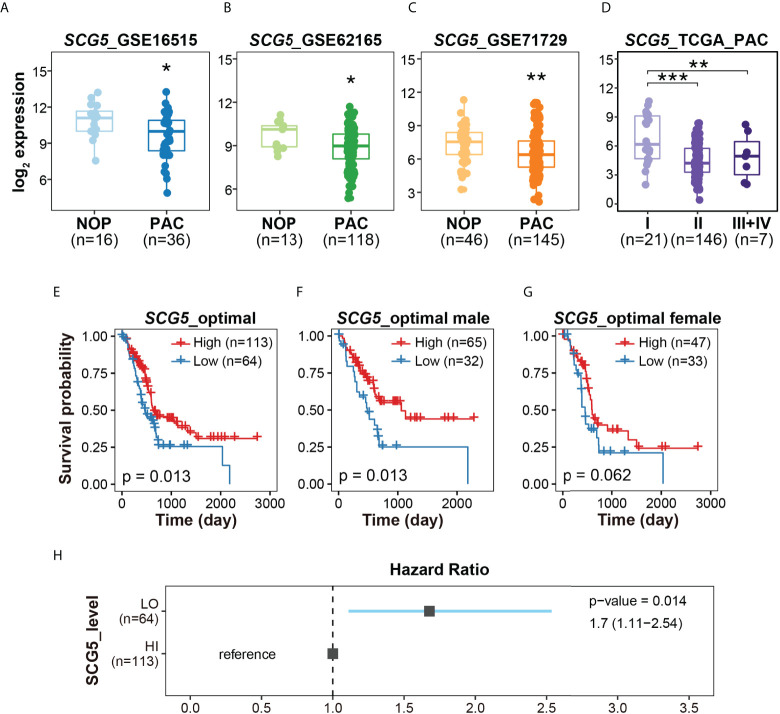
The expression profiles and clinicopathological signatures of *Secretogranin V* (*SCG5*). **(A–C)** Box plots presenting the levels of *SCG5* transcript in NOP and PAC. **(D)** Box plot showing *SCG5* expression at each stage of PAC. The boxplots **(A–D)** present the 25% quartile, the median, and 75% quartile. Student’s t- test **(A–C)** and one-way ANOVA **(D)** determined the statistical significance. ***p < 0.001; **p < 0.01; *p < 0.05. **(E–G)** Kaplan–Meier curves for overall survival of patients with PAC from TCGA cohort. **(H)** The forest plot showing the hazard ratio and 95% confidence intervals associated with the SCG5 level.

From each data set used in RF modeling, we investigated the expression patterns of *SCG5*, the RF-proposed potential biomarker. First, the normality of three data sets was estimated and taken as 0.1172 for GSE16515, 0.09327 for GSE62165, and 0.581 for GSE71729, following which the statistical significance was determined. As a result, *SCG5* expression levels in PAC were significantly lower (p = 0.014, 0.001, and 0.0092, respectively) than NOP, showcasing the potential of *SCG5* as a diagnostic marker (**Figures 3A–C**). We also investigated the correlation between *SCG5* expression and clinicopathological characteristics of PAC (i.e., TNM Classification of Malignant Tumors). The statistical significance among stages I, II, and III+IV of TCGA PAC data was also examined after the assessment of normality (p-value with Shapiro–Wilk test, 0.002716) (**Figure 3D**). It was observed that the expression level of *SCG5* at PAC stage I is substantially greater than in other stages, according to Tukey’s Honest Significant Difference test (a *post-hoc* test). In addition, we have compared pancreatic *SCG5* expression with individual the American Joint Committee on Cancer (AJCC) staging indices (**Supplementary Figure 1**). In the case of T stage, which is classified by the size of the tumor, the expression level of *SCG5* decreased significantly in T3 compared to T2. However, the comparisons between other stages failed due to the limitation of cohort size. For N stage, which is classified by the degree of spread to nearby lymph nodes, *SCG5* expression was statistically significantly lower in N1 compared to N0 but not in others, which is likely due to the N1b size limitation. In the case of the M stage, which is classified by the degree of metastasis, the number of samples in the M1 stage has a small N-power (n = 4), making it impossible to compare by stage. Although the results have the limitation of making a clear conclusion, this result, at least in part, confirms machine learning predictions and reveals that the expression level of *SCG5* reduces as the severity of the tumor grows, such as tumor size or lymph node metastasis.

To determine whether *SCG5* expression is correlated with prognosis of PAC, the survival probability was computed (**Figures 3E–H**). By first generating ROC curves as described in the “materials and methods” section, we were able to determine the optimal SCG5 expression values for each data set. According to the calculated optimal SCG5 expression level, groups of patients with high and low SCG5 expression levels were classified (high = 113 and low = 64). Overall, patients with high *SCG5* gene expression in PAC had a poor prognosis with low probability of survival. As a result, for 177 patients, higher *SCG5* expression signified higher survival rate, while lower *SCG5* expression signified lower survival rate, with a p-value of 0.013. In 97 male patients, the p-value was 0.013. Similarly, in 80 female patients, the p-value was 0.062. In line with the survival probability, the hazard ratio indicates that lower expression of pancreatic *SCG5* is associated with a poor prognosis of PAC (**Figures 3H**). Taken together, *SCG5* expression was lower in PAC compared with NOP (**Figures 3A–C**), lower in the early stages of PAC compared with the late stages (**Figure 3D**), and inversely associated with the overall survival rate (**Figure 3E**).

### Plasma SCG5 levels were lower in patients with PAC and associated with BMI and age

**Figure 4:**
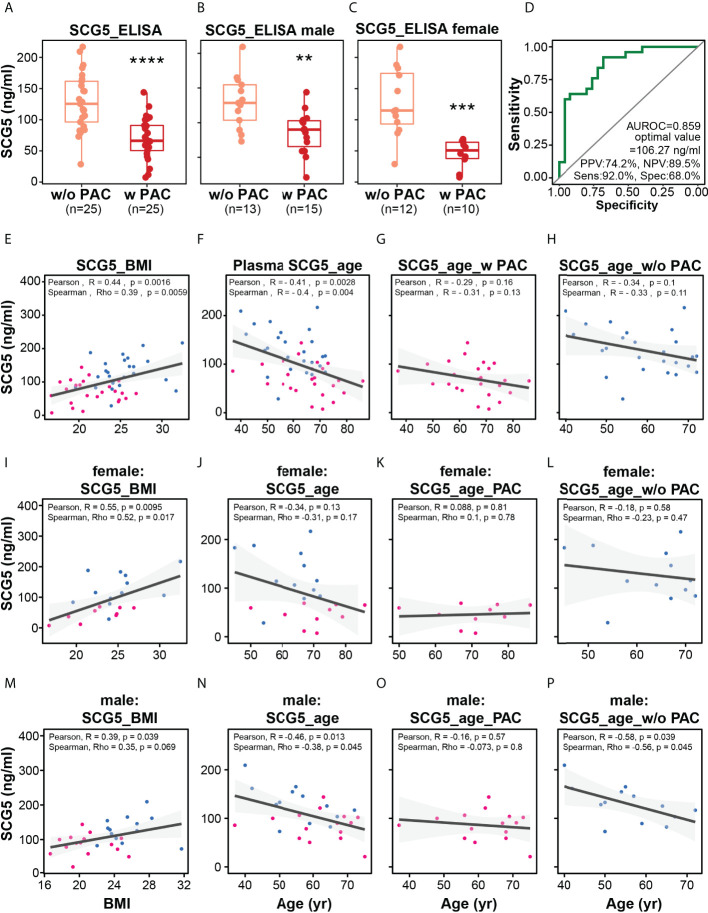
Plasma level of SCG5 distinguishes patients with PAC from those without PAC. **(A)** Plasma level of SCG5 in patients with PAC and those without PAC. **(B, C)** Plasma level in **(B)** male and **(C)** female participants. The boxplots **(A–C)** present the 25% quartile, the median, and 75% quartile. Student’s t- test computed the statistical significance. ****p < 0.0001, ***p < 0.001; **p < 0.01. **(D)** Receiver operating characteristic curve proposing optimal cut-off value (106.27 ng/ml) distinguishing patients with PAC from those without PAC. **(E–H)** Scatter plots estimating correlations between plasma SCG5 and BMI **(E)** or age **(F–H)**. **(I–O)** Scatter plots showing correlations between plasma SCG5 and BMI **(E)** or age in females **(I–L)** and males **(M–P)**.

Previously, we analyzed the correlation of *SCG5* expression with NOP and PAC in publicly available data as well as the relationship between *SCG5* expression and survival probability (or survival rate). Thereafter, using ELISA, we investigated SCG5 level in plasma samples collected from patients with and without PAC (w/o PAC). The samples’ normality was confirmed using the Shapiro-Wilk normality test, which yielded a p-value of 0.5949. Consequently, we computed the student’s t-test to ascertain the significance of plasma SCG5 level changes on sample status. Patients with PAC had significantly lower plasma SCG5 levels than those without PAC (w/o PAC mean = 128.7 ± 45.8; PAC mean = 69.7 ± 33.4; p = 4.9e-06). Similarly, plasma SCG5 levels in male (w/o PAC mean = 129.9 ± 38.1; PAC mean = 85.6 ± 30.5; p = 0.00269) and females (w/o PAC mean = 127.4 ± 54.7; PAC mean = 45.8 ± 22.0; p = 0.000268) were lower in patients with PAC compared with those w/o PAC (**Figures 4A–C**). To determine the optimal diagnostic threshold of SCG5 level in plasma that can distinguish between patients with and w/o PAC, an ROC curve was constructed. The optimal SCG5 value was 106.27 ng/ml (**Figure 4D**). Based on this value, the ability to distinguish between patients with and w/o PAC was 0.859 of AUROC, 74.2% of positive predictive value, 89.5% of negative predictive value, 92.0% of sensitivity, and 68.0% of specificity. To expand our understanding of the pathophysiology of SCG5, the correlation between its plasma level and clinical phenotypes (e.g., age, BMI, etc.) was then investigated. Interestingly, SCG5 levels in plasma were positively correlated with BMI (Pearson’s R = 0.44, p = 0.0016; Spearman’s Rho = 0.39, p = 0.0059) while negatively associated with age (Pearson’s R = -0.41, p = 0.0028; Spearman’s Rho = -0.4, p = 0.004) (**Figures 4E–H**). A mild negative association between plasma SCG5 level and age in both w/o PAC (Pearson’s R = -0.34, p =0.1; Spearman’s Rho = -0.33, p = 0.11) and PAC (Pearson’s R = -0.29, p =0.16; Spearman’s Rho = -0.31, p = 0.13) was observed although without any statistical significance. We also examined whether the correlations were gender independent (**Figure 4I–P**). Overall trends in females and males were generally consistent with the entire data set. However, probably due to the limitation of available sample size (number of patients), pancreatic *SCG5* expression was statistically associated with only BMI in either females or males. Together, in patients with PAC, plasma SCG5 levels were considerably lower than in those w/o PAC, demonstrating its diagnostic potential. Furthermore, we revealed that SCG5 levels in the plasma are associated with BMI and age.

### The area of subcutaneous adipocytes is positively correlated with *SCG5* expression in human pancreatic tissues

**Figure 5:**
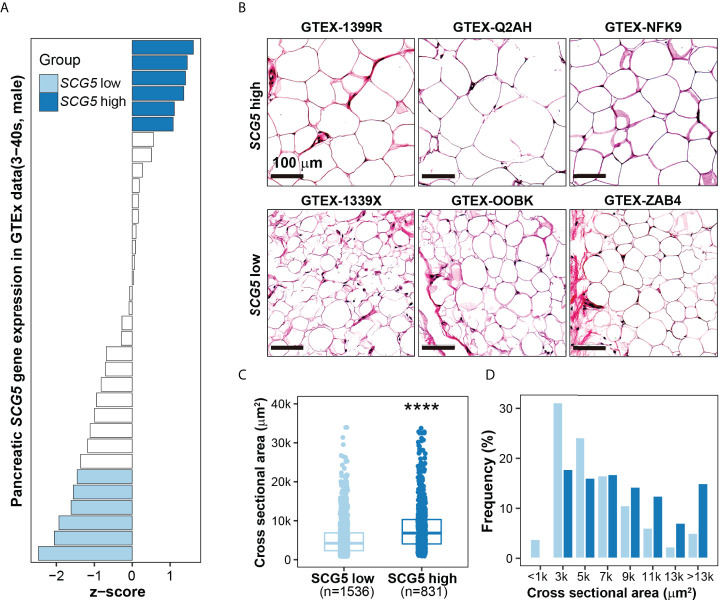
*SCG5* expression in human pancreatic tissues is positively associated with the size of subcutaneous adipocytes. **(A)** Bar plot showing pancreatic expression of *SCG5* in the human Genotype-Tissue Expression (GTEx) portal. **(B)** Hematoxylin and eosin-stained images of human subcutaneous adipose tissues from the GTEx portal, **(C, D)** Plots displaying the **(C)** size and **(D)** distribution of the cross-sectional area of adipocytes from H&E-stained histological images. The boxplots **(C)** present the 25% quartile, the median, and 75% quartile. Student’s *t*- test **(C)** assessed the statistical significance. ****p < 0.0001.

The aforementioned results imply that SCG5 has high potential as a marker for pancreatic cancer diagnosis and prognosis. Interestingly, we found that there is a correlation between *SCG5* expression levels and BMI. The incidence of CC is known to be high in pancreatic, stomach, lung, and colon cancers (12, 13). It affects around 80% of pancreatic cancer patients at some point in their illness (23, 24), implying that our findings may be associated with cachexia in pancreatic cancer, which is characterized by muscle and adipose atrophy in patients. Thus, we investigated how pancreatic *SCG5* is linked to adiposity. We hypothesized that if pancreatic SCG5 is a systemic humoral factor that influences adiposity (BMI), there would be a difference in adipocyte size between donors with high and low pancreatic *SCG5* expression. We conducted an integrative analysis of two omics data sets to evaluate this hypothesis (i.e., the pancreatic transcriptome and the histology of subcutaneous adipose tissue as a phenome) from the same donors in the GTEx database. We analyzed the pancreatic transcriptome of male (aged 30–40) samples to minimize age and gender biases because a negative correlation between *SCG5* expression and age was observed previously (**Figures 4F–H**). Pancreatic GTEx data samples were ordered according to *SCG5* expression levels and 12 were chosen from the top (n = 6) and bottom (n = 6), accounting for roughly one-third of all samples (**Figure 5A**). We downloaded hematoxylin and eosin-stained histological images of SAT for the selected samples and measured the cell size. Assessment of the CSA of human SAT determined that higher level of pancreatic *SCG5* is associated with larger cell size in human SAT (**Figure 5B**). The quantification of CSA and frequency of cell size in SAT further validated the hypothesis (**Figure 5C, D**). This result indicates that pancreatic *SCG5* expression is positively associated with the size of subcutaneous adipocytes in humans. Aligning with the result showing positive correlation between plasma SCG5 and BMI (adiposity, **Figure 4E**), this implies that *SCG5* may be a systemic regulator of adipose tissue homeostasis and may contribute to cachexia in patients with PAC.

### Unbiased transcriptome analysis connected pancreatic SCG5 expression to the adipopenia phenotype

Our findings imply that pancreatic SCG5 could be a systemic humoral factor that contributes to adiposity or adipopenia. This prompted us to explore further in order to find any concealed the downstream mechanism of SCG5 in human SAT. Thus, we attempted to comprehend the phenomena of positive correlation between pancreatic *SCG5* expression and adipocyte size or adiposity (BMI) at the molecular level by performing an unbiased gene set enrichment analysis, a gold standard for investigating an associated gene set, signaling pathway, or genes associated to diseases in humans (29, 36).

**Figure 6:**
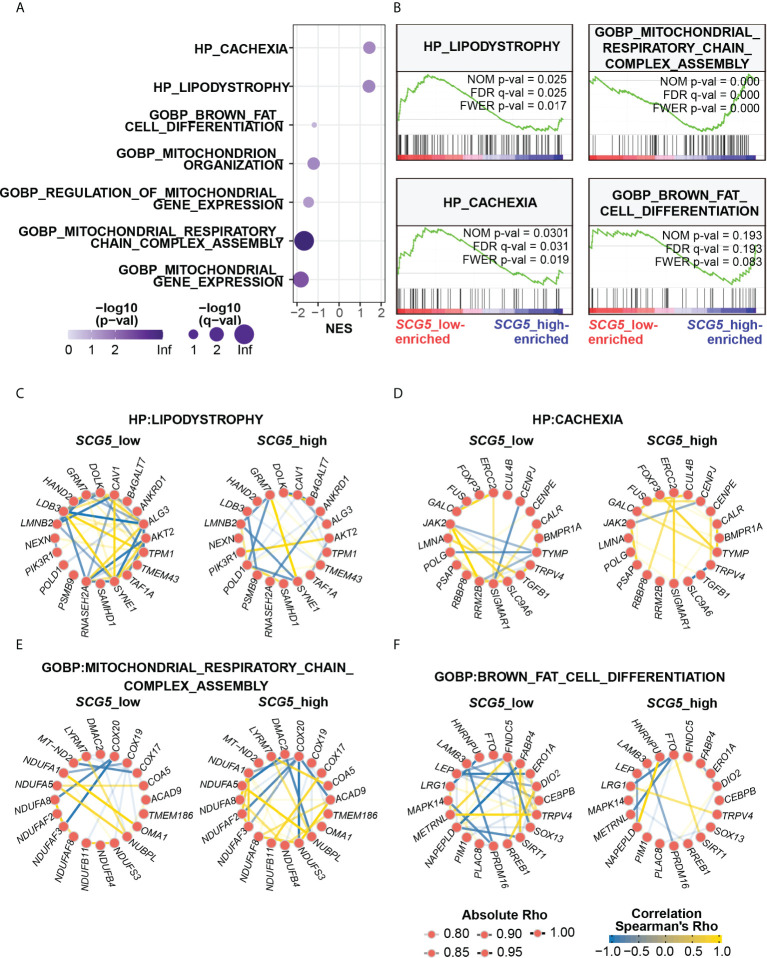
Unbiased transcriptomic analysis shows that pancreatic *SCG5* expression is associated with the adipopenia phenotype. **(A)** Bubble plot summarizing the results of gene set enrichment analysis (GSEA) dissecting adipose transcriptomic profiles from donors of pancreatic *SCG5*-high or -low groups. **(B)** Representative enrichment plots of cachexia and adipopenia-related gene sets. **(C–F)** Gene networks comparing correlations among top genes of each indicated gene set, human phenotype ontology (HP) Lipodystrophy **(C)**, HP Cachexia **(D)**, Gene Ontology biological process (GOBP) Mitochondrial respiratory chain complex assembly **(E)**, and GOBP Brown fat cell differentiation **(F)**.

SAT transcriptomic data were prepared in the same way as done for adipocyte size analysis (**Figure 5**) for GSEA. GSEA was performed by dividing the data into two groups (i.e., *SCG5*-high, and *SCG5*-low) according to *SCG5* expression level in the pancreas. To analyze the associated biological processes and phenotypes in SAT, we used two complete gene sets: Gene Ontology Biological Process (GOBP) and Human Phenotype Ontology (HP). GSEA revealed seven top-correlated gene sets that may be associated with cachexia, adipopenia, or adiposity (**Figure 6A**). Interestingly, GSEA also revealed two gene sets of HP cachexia (HP:0004326) and HP lipodystrophy (HP:0009125) that were significantly associated to pancreatic *SCG5* expression in SAT. Moreover, gene sets of GOBP brown fat cell differentiation (GO:0050873), GOBP mitochondrion organization (GO:0007005), GOBP regulation of mitochondrial gene expression (GO:0062125), GOBP mitochondrial respiratory chain complex assembly (GO:0033108), and GOBP mitochondrial gene expression (GO:0140053) were also associated with pancreatic SCG5 expression. It is reported that brown fat cell activity, browning of adipose tissue, and mitochondria function may be associated with cachexia (37–39).

Since each gene set contains numerous genes, we performed gene network analysis (31) to analyze how genes in each gene set are associated with pancreatic *SCG5* expression. Gene networks were constructed using ten top- and bottom-ranked genes in the respective gene sets. In HP lipodystrophy, the pancreatic *SCG5*-low donors had stronger and more correlations among lipodystrophy genes than pancreatic *SCG5*-high donors, and positive correlation between *GRM7* and *SYNBE1* was stronger in pancreatic *SCG5*-high donors than *SCG5*-low donors (**Figure 6C**). In HP cachexia, the number or degree of the overall correlation between *SCG5*-low and *SCG5*-high donors did not differ significantly. However, some of the negative correlations dispersed, reappeared, and became stronger, such as *CENPJ*-*SIGMAR1*, *CENPJ*-*JAK2*, and *SLC9A6*-*TRPV4* in *SCG5*-high donors, while some positive correlations became stronger, such as *ERCC2*-*SIGMAR1*, *TYMP*-*FOXP3*, and *TYMP*-*GALC* (**Figure 6D**). In GOBP mitochondrial respiratory chain complex assembly, *SCG5*-high donors had stronger and more correlations than *SCG5*-low donors. Negative correlation, such as *DMAC2*-*NUBPL*, was stronger in *SCG5*-high donors than in *SCG5*-low donors, although negative correlation such as *COX20*-*NDUFA8* was attenuated (**Figure 6E**). In GOBP brown fat cell differentiation, *SCG5*-low donors demonstrated stronger and many more correlations than *SCG5*-high donors. The weaker correlation between *LRG1* and *TRPV4* in *SCG5*-low donors was found to be strong in *SCG5*-high donors (**Figure 6F**). Altogether, GSEA analysis revealed that genes and gene sets involved in adipose tissue wasting and mitochondrial activity were tightly associated with pancreatic *SCG5* expression level. This signifies that SCG5 may be a systemic humoral factor and may contribute to cachexia in pancreatic cancer, which should be verified further.

### The effect of recombinant SCG5 protein on adipocyte biology

Our findings indicate that *SCG5* expression in PAC tissue and SCG5 level in blood are associated with adiposity (e.g., BMI) and can be a good biomarker for recognizing patients with PAC and predicting their prognosis. To determine whether SCG5 protein alone can directly affect adipocyte biology (e.g., differentiation or lipid accumulation), three different concentrations of rSCG5 were treated during and after differentiation of 3T3L-1 adipocytes. First, we selected three different concentrations (50, 100, and 200 ng/ml) based on the plasma concentration that we measured from the human donors (**Figure 4**). The effect of rSCG5 was determined with Oil-Red-O, which stains neutral triglycerides and lipids (40). The accumulation of neutral lipid did not change significantly across all three concentrations of rSCG5 (**Supplementary Figures 2A–D**). Consistently, the patterns of gene expression involved in adipocyte biology, including *Srebf1, Pparg, Adipoq, Fasn, Scd1, Acc1, Slc2a4/Glut4, Cpt1a, Acadm, Acadvl, and Ascsl1*, were not changed significantly in all tested conditions (**Supplementary Figures 2E, F**), implying rSCG5 may not work as a signaling ligand on adipocytes.

## Discussion

In this study, using supervised machine learning models, we discovered that *SCG5* gene can distinguish between NOP and PAC as well as short-lived and long-lived patients with PAC. We validated the difference in plasma SCG5 levels between w/o PAC and PAC using ELISA and proposed the diagnostic threshold (106.27 ng/ml) in our cohort (**Figures 4A–D**), which needs to be further tested in an independent large cohort to overcome our race and geological biases. In addition, we were unable to validate the potential of *SCG5* as a prognostic marker in plasma samples due to limited cohort size and lack of information, such as data regarding survival days. The power of *SCG5* as a prognostic marker should be examined together with the evaluation of any correlation between plasma SCG5 levels and clinicopathological characteristics (TNM stage, survival rate, etc.) in an independent cohort with PAC. Our results show that the calculated survival rate indicates that *SCG5* expression can be a prognostic marker for male patients but not for females (**Figures 3E–G**). However, plasma SCG5 level was much strongly reduced in female patients compared with male patients (**Figures 4B, C**), implying that plasma SCG5 levels show gender bias. It is hard to explain why the trends of pancreatic SCG5 mRNA and plasma SCG5 protein are inconsistent. (**Figures 3F, G**, **4B, C**). There are many possibilities. The gender bias could be originated by (1) the cohort size limitation; (2) the genetic background limitation (ELISA was performed only on the Korean population, but TCGA data was primarily composed of Caucasians (n = 162), with other races limited to Asians (n = 11), African Americans (n = 7), and unreported (n = 5), and so on); (3) the mismatch between transcription rate (mRNA) and translation (protein) rat; and (4) so on. This gender significance of SCG5 level in PAC should be investigated further in an independent larger cohort to confirm a potential gender effect.

The analysis showing the degree of association between SCG5 plasma protein and BMI also demonstrates that this association exists not only in cancer patients but also in donors without cancer, implying that plasma SCG5 has a role in both physiology and pathology (**Figure 4E**). This relationship is consistent in both genders (**Figures 4I, M**). In addition, the level of SCG5 expression was lower in pancreatic cancer patients with low BMI (**Figure 5E**), although it is unclear whether SCG5 induces cachexia or adipopenia. In order to verify the association of plasma SCG5 with pathology (e.g., cachexia, adipopenia, lipodystrophy, obesity, etc.) and physiology (e.g., body weight, BMI, gender, race, etc.), it will be valuable to explore the association of SCG5 with pathophysiological factors in a large population to overcome any bias linked to genetic and geological backgrounds.

Furthermore, to discover the mechanism-of-action and biological function of SCG5, GSEA was performed on gene expression data from subcutaneous adipose tissue samples with high and low pancreatic *SCG5* expression. We found association in gene sets related to adipose tissue wasting phenotype such as cachexia, lipodystrophy, mitochondrial function, and brown fat cell differentiation (**Figure 6**), which is consistent with the CSA analysis of SAT (**Figure 5**). Although a few studies have reported no correlation between cachexia and brown adipose tissue (41), many studies have found a link2 between cachexia and the activity of brown adipose tissue and the browning of white fat (37–39). Mitochondrial function and homeostasis are an essential tissue for brown adipose tissue, and its function is fully dependent on mitochondrial function (42–44). Several studies have proposed that cancer-originating humoral factors drive dysregulation of mitochondria, including their hyperactivation (18, 45–48). The genes included in lipodystrophy are directly associated with adipose tissue development, homeostasis, lipogenesis, and lipolysis. This implies that alteration in genes that can directly affect adipocyte size is systematically related to alteration in pancreatic SCG5.

Although SCG5 has obvious promise as a diagnostic and prognostic biomarker for PAC, we found no significant effect of rSCG5 on adipocyte biology (**Supplementary Figure 2**), demonstrating that SCG5 cannot directly govern adipose tissue. The molecular function of SCG5, also known as P7B2, is known to prevent the aggregation of other secreted proteins as a secreted chaperone protein (49), indicating that SCG5 might not work as a ligand but rather it may facilitate other protein ligands in the blood. Interestingly, genetic analysis using expression quantitative trait loci (a.k.a. eQTL) of SCG5 also reported that pituitary levels of SCG5 were correlated inversely with body weight in mice (50), consistently supporting our observation of inverse correlations between plasma SCG5 and human BMI and adiposity. The function of blood SCG5 in adipocyte biology should be investigated *in vivo* rather than *in vitro*, first. Then, both proteins should be evaluated on cultured adipocytes after the deconvolution of a plasma partner protein, facilitated by SCG5, a blood chaperone protein. Taken together, our findings demonstrate that RF- and XGBoost-featured *SCG5* is a circulating diagnostic indicator of PAC that may be a pathophysiological and systemic regulator for adiposity and can be a novel therapeutic target for both adipose tissue-wasting (e.g., cachexia, adipopenia, lipodystrophy, etc.) and accumulating diseases (e.g., obesity).

## Data availability statement

The original contributions presented in the study are included in the article/**Supplementary Material**. Further inquiries can be directed to the corresponding authors.

## Ethics statement

The studies involving human participants were reviewed and approved by This study was approved by the Institutional Review Board of Chungnam National University Hospital (CNUH 2019-11-043), which waived the requirement for informed consent. The patients/participants provided their written informed consent to participate in this study.

## Author contributions

YJ, M-KY, H-SY, and DR conceived and designed the project. YJ performed all analyses, including machine learning modeling. M-KY and H-SY designed and conducted human study including analysis of clinical manifestations and diagnosis. TD and M-KY performed histological analysis. JK performed the enzyme linked immunosorbent assay. H-SY and DR supervised the projects. YJ and DR wrote the manuscript with technical support of the co-authors. All authors contributed to the article and approved the submitted version.

## Funding

D.R. was supported by grants from the National Research Foundation of Korea (NRF) funded by the Korean government (NRF-2020R1A2C2010964 and 2021R1A5A8029876). H-S.Y. was supported by the NRF (NRF- 2021R1A2C4001829 and 2022M3A9B6017654) and Korea Research Institute of Bioscience and Biotechnology Research Initiative Program (KGM9992211). Y.J., M-K. Y. and J.K. were supported by the Basic Science Research Program of the NRF funded by the Ministry of Education (2022R1I1A1A01063460 to Y.J., 2017R1D1A1B04031187 to M-K.Y. and 2020R1I1A1A01074737 to J.K.).

## Acknowledgments

The authors like to thank all the participants of the study for blood donation and the members of the Ryu Lab and Yi lab for constructive discussions and technical support.

## Conflict of interest

The authors declare that the research was conducted in the absence of any commercial or financial relationships that could be construed as a potential conflict of interest.

## Publisher’s note

All claims expressed in this article are solely those of the authors and do not necessarily represent those of their affiliated organizations, or those of the publisher, the editors and the reviewers. Any product that may be evaluated in this article, or claim that may be made by its manufacturer, is not guaranteed or endorsed by the publisher.

## Abbreviations

PAC, Pancreatic adenocarcinoma; CC, Cancer cachexia; NOP, normal human pancreas; TCGA, The Cancer Genome Atlas; SCG5, Secretogranin V; GTEx, Genotype-Tissue Expression; BMI, body mass index; RF, Random Forest; AUROC, area under the receiver operating characteristics; ELISA, enzyme-linked immunosorbent assay; SAT, subcutaneous adipose tissue; CSA, cross sectional area; GSEA, Gene Set Enrichment Analysis; GOBP, Gene Ontology Biological Process; HP, Human Phenotype Ontology.

## References

[1] Sung H Ferlay J Siegel RL Laversanne M Soerjomataram I Jemal A. Global cancer statistics 2020: GLOBOCAN estimates of incidence and mortality worldwide for 36 cancers in 185 countries. CA Cancer J Clin (2021) 71(3):209–49. doi: 10.3322/caac.21660 33538338

[2] Wang S Zheng Y Yang F Zhu L Zhu XQ Wang ZF. The molecular biology of pancreatic adenocarcinoma: translational challenges and clinical perspectives. Signal Transduct Target Ther (2021) 6(1):249. doi: 10.1038/s41392-021-00659-4 34219130 PMC8255319

[3] Ni XG Bai XF Mao YL Shao YF Wu JX Shan Y. The clinical value of serum CEA, CA19-9, and CA242 in the diagnosis and prognosis of pancreatic cancer. Eur J Surg Oncol (2005) 31(2):164–9. doi: 10.1016/j.ejso.2004.09.007 15698733

[4] Safi F Roscher R Bittner R Schenkluhn B Dopfer HP Beger HG. High sensitivity and specificity of CA 19-9 for pancreatic carcinoma in comparison to chronic pancreatitis. serological and immunohistochemical findings. Pancreas (1987) 2(4):398–403. doi: 10.1097/00006676-198707000-00006 3306667

[5] Bilchik A Miyashiro M Kelley M Kuo C Fujiwara Y Nakamori S. Molecular detection of metastatic pancreatic carcinoma cells using a multimarker reverse transcriptase-polymerase chain reaction assay. Cancer (2000) 88(5):1037–44. doi: 10.1002/(sici)1097-0142(20000301)88:5<1037::aid-cncr13>3.0.co;2-h 10699892

[6] Haglund C Lundin J Kuusela P Roberts PJ. CA 242, a new tumour marker for pancreatic cancer: a comparison with CA 19-9, CA 50 and CEA. Br J Cancer (1994) 70(3):487–92. doi: 10.1038/bjc.1994.332 PMC2033366 8080735

[7] Carpelan-Holmstrom M Haglund C Kuusela P Jarvinen H Roberts PJ. Preoperative serum levels of CEA and CA 242 in colorectal cancer. Br J Cancer (1995) 71(4):868–72. doi: 10.1038/bjc.1995.167 PMC2033721 7710956

[8] Dou H Sun G Zhang L. CA242 as a biomarker for pancreatic cancer and other diseases. Prog Mol Biol Transl Sci (2019) 162:229–39. doi: 10.1016/bs.pmbts.2018.12.007 30905452

[9] Arrieta O Villarreal-Garza C Martinez-Barrera L Morales M Dorantes-Gallareta Y Pena-Curiel O. Usefulness of serum carcinoembryonic antigen (CEA) in evaluating response to chemotherapy in patients with advanced non small-cell lung cancer: a prospective cohort study. BMC Cancer (2013) 13:254. doi: 10.1186/1471-2407-13-254 23697613 PMC3665670

[10] Fukuda I Yamakado M Kiyose H. Influence of smoking on serum carcinoembryonic antigen levels in subjects who underwent multiphasic health testing and services. J Med Syst (1998) 22(2):89–93. doi: 10.1023/a:1022643102208 9571515

[11] Evans WJ Morley JE Argiles J Bales C Baracos V Guttridge D. Cachexia: a new definition. Clin Nutr (2008) 27(6):793–9. doi: 10.1016/j.clnu.2008.06.013 18718696

[12] Baracos VE Martin L Korc M Guttridge DC Fearon KCH. Cancer-associated cachexia. Nat Rev Dis Primers (2018) 4:17105. doi: 10.1038/nrdp.2017.105 29345251

[13] Tan CR Yaffee PM Jamil LH Lo SK Nissen N Pandol SJ. Pancreatic cancer cachexia: a review of mechanisms and therapeutics. Front Physiol (2014) 5:88. doi: 10.3389/fphys.2014.00088 24624094 PMC3939686

[14] Fearon K Strasser F Anker SD Bosaeus I Bruera E Fainsinger RL. Definition and classification of cancer cachexia: an international consensus. Lancet Oncol (2011) 12(5):489–95. doi: 10.1016/S1470-2045(10)70218-7 21296615

[15] Argiles JM Busquets S Stemmler B Lopez-Soriano FJ. Cancer cachexia: understanding the molecular basis. Nat Rev Cancer (2014) 14(11):754–62. doi: 10.1038/nrc3829 25291291

[16] Kir S White JP Kleiner S Kazak L Cohen P Baracos VE. Tumour-derived PTH-related protein triggers adipose tissue browning and cancer cachexia. Nature (2014) 513(7516):100–4. doi: 10.1038/nature13528 PMC4224962 25043053

[17] Yeom E Yu K. Understanding the molecular basis of anorexia and tissue wasting in cancer cachexia. Exp Mol Med (2022) 54(4):426–432. doi: 10.1038/s12276-022-00752-w PMC9076846 35388147

[18] Fukawa T Yan-Jiang BC Min-Wen JC Jun-Hao ET Huang D Qian CN. Excessive fatty acid oxidation induces muscle atrophy in cancer cachexia. Nat Med (2016) 22(6):666–71. doi: 10.1038/nm.4093 27135739

[19] Dao T Green AE Kim YA Bae SJ Ha KT Gariani K. Sarcopenia and muscle aging: A brief overview. Endocrinol Metab (Seoul) (2020) 35(4):716–32. doi: 10.3803/EnM.2020.405 PMC7803599 33397034

[20] Bing C Trayhurn P. New insights into adipose tissue atrophy in cancer cachexia. Proc Nutr Soc (2009) 68(4):385–92. doi: 10.1017/S0029665109990267 19719894

[21] Bachmann J Heiligensetzer M Krakowski-Roosen H Buchler MW Friess H Martignoni ME. Cachexia worsens prognosis in patients with resectable pancreatic cancer. J Gastrointest Surg (2008) 12(7):1193–201. doi: 10.1007/s11605-008-0505-z 18347879

[22] Anandavadivelan P Lagergren P. Cachexia in patients with oesophageal cancer. Nat Rev Clin Oncol (2016) 13(3):185–98. doi: 10.1038/nrclinonc.2015.200 26573424

[23] Poulia KA Sarantis P Antoniadou D Koustas E Papadimitropoulou A Papavassiliou AG. Pancreatic cancer and cachexia-metabolic mechanisms and novel insights. Nutrients (2020) 12(6):1543. doi: 10.3390/nu12061543 PMC7352917 32466362

[24] Fearon KC Voss AC Hustead DS Cancer Cachexia Study G. Definition of cancer cachexia: effect of weight loss, reduced food intake, and systemic inflammation on functional status and prognosis. Am J Clin Nutr (2006) 83(6):1345–50. doi: 10.1093/ajcn/83.6.1345 16762946

[25] Zhang ZM Wang JS Zulfiqar H Lv H Dao FY Lin H. Early diagnosis of pancreatic ductal adenocarcinoma by combining relative expression orderings with machine-learning method. Front Cell Dev Biol (2020) 8:582864. doi: 10.3389/fcell.2020.582864 33178697 PMC7593596

[26] Camacho DM Collins KM Powers RK Costello JC Collins JJ. Next-generation machine learning for biological networks. Cell (2018) 173(7):1581–92. doi: 10.1016/j.cell.2018.05.015 29887378

[27] Ibrahim S Nazir S Velastin SA. Feature selection using correlation analysis and principal component analysis for accurate breast cancer diagnosis. J Imaging (2021) 7(11):225. doi: 10.3390/jimaging7110225 34821856 PMC8625715

[28] Ji M Jo Y Choi SJ Kim SM Kim KK Oh B-C. Plasma metabolomics profiling and machining learning-driven prediction of nonalcoholic steatohepatitis. medRxiv (2021) 2021:21265434. doi: 10.1101/2021.10.24.21265434

[29] Chung H Jo Y Ryu D Jeong C Choe SK Lee J. Artificial-intelligence-driven discovery of prognostic biomarker for sarcopenia. J Cachexia Sarcopenia Muscle (2021) 12(6):2220–30. doi: 10.1002/jcsm.12840 PMC8718042 34704369

[30] Uhlen M Karlsson MJ Hober A Svensson AS Scheffel J Kotol D. The human secretome. Sci Signal (2019) 12(609):eaaz0274. doi: 10.1126/scisignal.aaz0274 31772123

[31] Kim J Lee H Jin EJ Jo Y Kang BE Ryu D. A microfluidic device to fabricate one-step cell bead-laden hydrogel struts for tissue engineering. Small (2022) 18(1):e2106487. doi: 10.1002/smll.202106487 34854561

[32] Oppi S Nusser-Stein S Blyszczuk P Wang X Jomard A Marzolla V. Macrophage NCOR1 protects from atherosclerosis by repressing a pro-atherogenic PPARgamma signature. Eur Heart J (2020) 41(9):995–1005. doi: 10.1093/eurheartj/ehz667 31529020

[33] Li L Zhang JW Jenkins G Xie F Carlson EE Fridley BL. Genetic variations associated with gemcitabine treatment outcome in pancreatic cancer. Pharmacogenet Genomics (2016) 26(12):527–37. doi: 10.1097/FPC.0000000000000241 PMC5083195 27749787

[34] Janky R Binda MM Allemeersch J Van den Broeck A Govaere O Swinnen JV. Prognostic relevance of molecular subtypes and master regulators in pancreatic ductal adenocarcinoma. BMC Cancer (2016) 16:632. doi: 10.1186/s12885-016-2540-6 27520560 PMC4983037

[35] Moffitt RA Marayati R Flate EL Volmar KE Loeza SG Hoadley KA. Virtual microdissection identifies distinct tumor- and stroma-specific subtypes of pancreatic ductal adenocarcinoma. Nat Genet (2015) 47(10):1168–78. doi: 10.1038/ng.3398 PMC4912058 26343385

[36] Subramanian A Tamayo P Mootha VK Mukherjee S Ebert BL Gillette MA. Gene set enrichment analysis: a knowledge-based approach for interpreting genome-wide expression profiles. Proc Natl Acad Sci U.S.A. (2005) 102(43):15545–50. doi: 10.1073/pnas.0506580102 PMC1239896 16199517

[37] Kir S Spiegelman BM. Cachexia & brown fat: A burning issue in cancer. Trends Cancer (2016) 2(9):461–3. doi: 10.1016/j.trecan.2016.07.005 PMC5407404 28459108

[38] Sun X Feng X Wu X Lu Y Chen K Ye Y. Fat wasting is damaging: Role of adipose tissue in cancer-associated cachexia. Front Cell Dev Biol (2020) 8:33. doi: 10.3389/fcell.2020.00033 32117967 PMC7028686

[39] Petruzzelli M Schweiger M Schreiber R Campos-Olivas R Tsoli M Allen J. A switch from white to brown fat increases energy expenditure in cancer-associated cachexia. Cell Metab (2014) 20(3):433–47. doi: 10.1016/j.cmet.2014.06.011 25043816

[40] Kim K Ryu D Dongiovanni P Ozcan L Nayak S Ueberheide B. Degradation of PHLPP2 by KCTD17, *via* a glucagon-dependent pathway, promotes hepatic steatosis. Gastroenterology (2017) 153(6):1568–1580 e10. doi: 10.1053/j.gastro.2017.08.039 28859855 PMC5705280

[41] Becker AS Zellweger C Bacanovic S Franckenberg S Nagel HW Frick L. Brown fat does not cause cachexia in cancer patients: A large retrospective longitudinal FDG-PET/CT cohort study. PloS One (2020) 15(10):e0239990. doi: 10.1371/journal.pone.0239990 33031379 PMC7544086

[42] Nicholls DG Locke RM. Thermogenic mechanisms in brown fat. Physiol Rev (1984) 64(1):1–64. doi: 10.1152/physrev.1984.64.1.1 6320232

[43] Yuko OO Saito M. Brown fat as a regulator of systemic metabolism beyond thermogenesis. Diabetes Metab J (2021) 45(6):840–52. doi: 10.4093/dmj.2020.0291 PMC8640153 34176254

[44] Hittelman KJ Lindberg O Cannon B. Oxidative phosphorylation and compartmentation of fatty acid metabolism in brown fat mitochondria. Eur J Biochem (1969) 11(1):183–92. doi: 10.1111/j.1432-1033.1969.tb00759.x 5353600

[45] Beltra M Pin F Ballaro R Costelli P Penna F. Mitochondrial dysfunction in cancer cachexia: Impact on muscle health and regeneration. Cells (2021) 10(11):3150. doi: 10.3390/cells10113150 34831373 PMC8621344

[46] VanderVeen BN Fix DK Carson JA. Disrupted skeletal muscle mitochondrial dynamics, mitophagy, and biogenesis during cancer cachexia: A role for inflammation. Oxid Med Cell Longev (2017) 2017:3292087. doi: 10.1155/2017/3292087 28785374 PMC5530417

[47] Brown JL Rosa-Caldwell ME Lee DE Blackwell TA Brown LA Perry RA. Mitochondrial degeneration precedes the development of muscle atrophy in progression of cancer cachexia in tumour-bearing mice. J Cachexia Sarcopenia Muscle (2017) 8(6):926–38. doi: 10.1002/jcsm.12232 PMC5700433 28845591

[48] de Castro GS Simoes E Lima J Ortiz-Silva M Festuccia WT Tokeshi F. Human cachexia induces changes in mitochondria, autophagy and apoptosis in the skeletal muscle. Cancers (Basel) (2019) 11(9):1264. doi: 10.3390/cancers11091264 PMC6770124 31466311

[49] Gabreels BA Swaab DF de Kleijn DP Seidah NG Van de Loo JW Van de Ven WJ. Attenuation of the polypeptide 7B2, prohormone convertase PC2, and vasopressin in the hypothalamus of some prader-willi patients: indications for a processing defect. J Clin Endocrinol Metab (1998) 83(2):591–9. doi: 10.1210/jcem.83.2.4542 9467579

[50] Farber CR Chitwood J Lee SN Verdugo RA Islas-Trejo A Rincon G. Overexpression of Scg5 increases enzymatic activity of PCSK2 and is inversely correlated with body weight in congenic mice. BMC Genet (2008) 9:34. doi: 10.1186/1471-2156-9-34 18439298 PMC2386500

