# Supplementary material for: Machine learning-featured Secretogranin V is a circulating diagnostic biomarker for pancreatic adenocarcinomas associated with adipopenia
Source: Front Oncol. 2022 Aug 17;12:942774. doi: 10.3389/fonc.2022.942774 (PMC9428794; doi:10.3389/fonc.2022.942774)
Supplement: Supplementary file 1 [file DataSheet_1.docx]

SUPPLEMENTARY INFORMATION

Machine learning-featured Secretogranin V is a circulating diagnostic biomarker for pancreatic adenocarcinomas associated with adipopenia

Yunju Jo^1^, Min-Kyung Yeo^2^, Tam Dao^1^, Jeongho Kwon^1^, Hyon‐Seung Yi^3,4^,
Dongryeol Ryu^1^


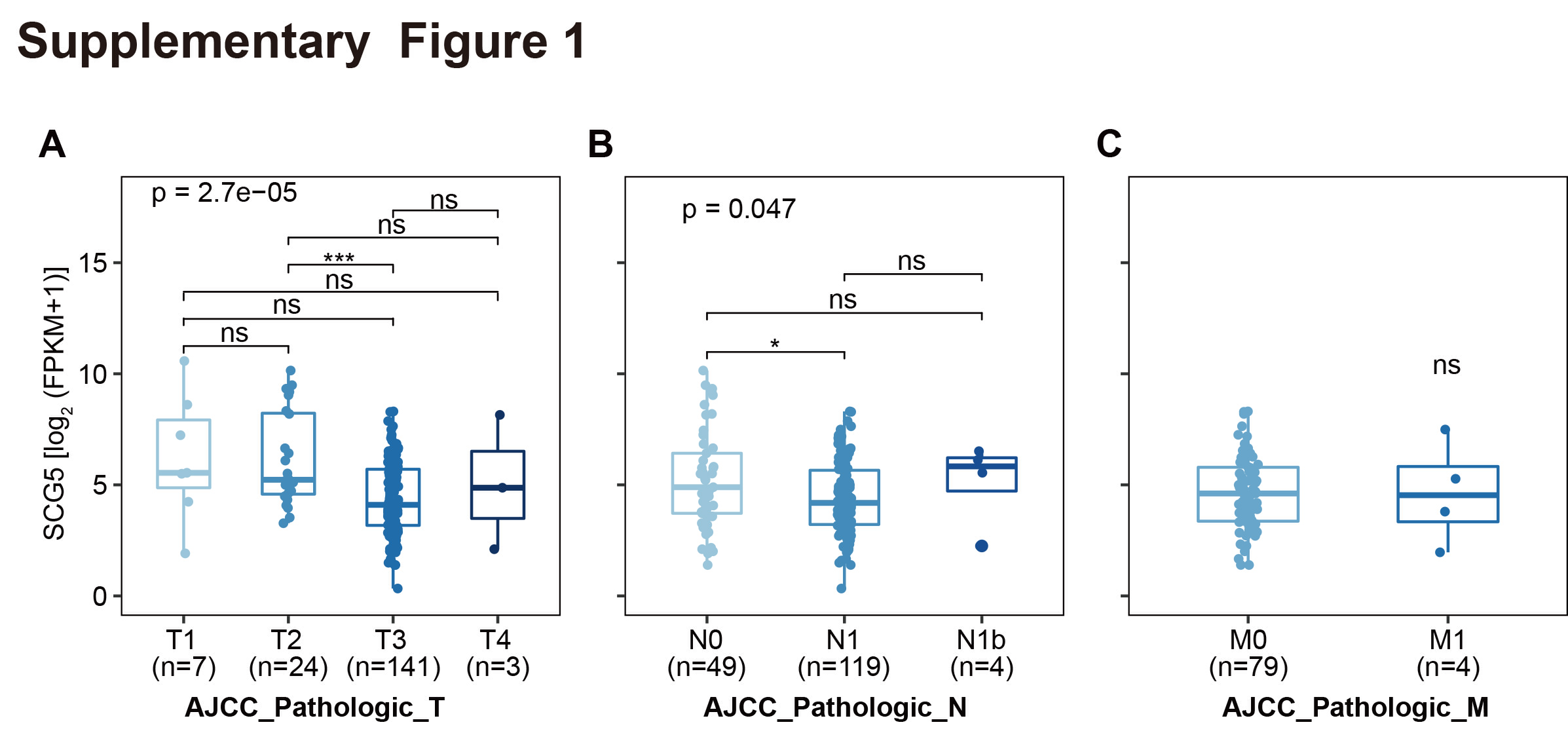


**SUPPLEMENTARY FIGURE 1. Boxplots showing the association between pancreatic *SCG5* expression and the American Joint Committee on Cancer (AJCC) pathological stages. (A-C)** Box plots presenting the levels of *SCG5* transcript in AJCC T, N, and M stages. The boxplots present the 25% quartile, the median, and 75% quartile. Student's *t*- test (between two groups) and one-way ANOVA (among three or more groups) determined the statistical significance. ***, p < 0.001; *, p < 0.05.


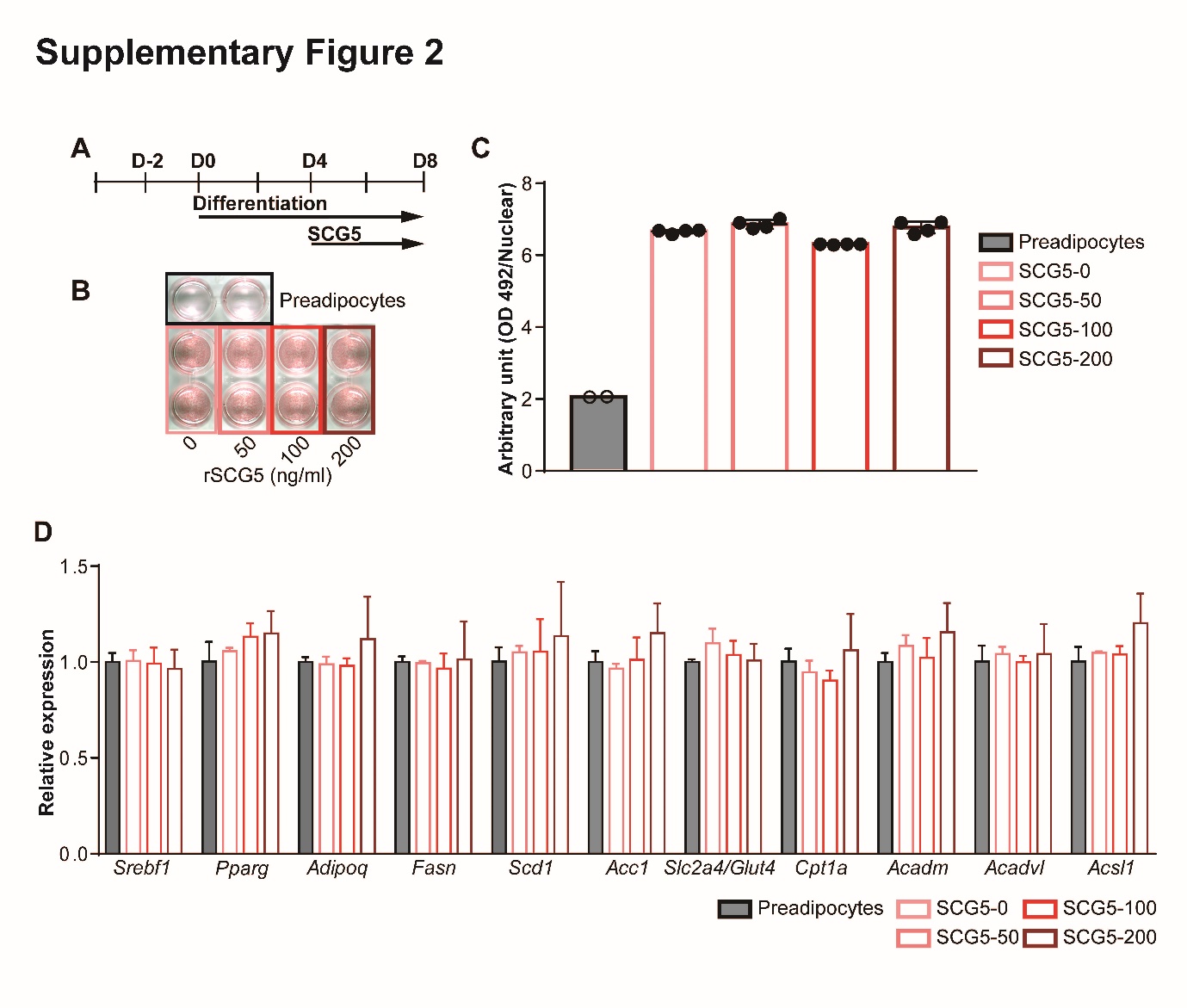


**SUPPLEMENTARY FIGURE 2. The effect of recombinant SCG5 protein (rSCG5) on adipocyte biology. (A-D)** Oil-Red-O stain showing the amount of neutral lipids in 3T3L-1 adipocytes. (A, C) Schematic diagram summarizing the experimental timeline. (B, D) Boxplots showing the intracellular amount of neutral lipids. (E, F) Boxplots showing the expression profiles of genes involve in adipocyte biology. The boxplots present the 25% quartile, the median, and 75% quartile. Student's t- test determined the statistical significance. ***, p < 0.001; **, p < 0.01; *, p < 0.05.

**SUPPLEMENTARY TABLE 1. The oligo list for qRT-PCR**

| **Gene** | **Forward primer (5’ - 3’)** | **Reverse primer (5’ - 3’)** |
| --- | --- | --- |
| *Srebf1* | GATGTGCGAACTGGACACAG | CATAGGGGGCGTCAAACAG |
| *Pparg1* | ACGTTCTGACAGGACTGTGTGAC | TGATTCCGAAGTTGGTGGGCC |
| *Adipoq* | GAACTTGTGCAGGTTGGATGGC | CCTGTCTCACCCTTAGGACCAA |
| *Fasn* | GGAGGTGGTGATAGCCGGTAT | TGGGTAATCCATAGAGCCCAG |
| *Scd1* | TTCTTGCGATACACTCTGGTGC | CGGGATTGAATGTTCTTGTCGT |
| *Acc1* | ATGGGCGGAATGGTCTCTTTC | TGGGGACCTTGTCTTCATCAT |
| *Glut4* | GTGACTGGAACACTGGTCCTA | CCAGCCACGTTGCATTGTAG |
| *Acc1* | ATGGGCGGAATGGTCTCTTTC | TGGGGACCTTGTCTTCATCAT |
| *Acc2* | CCTTTGGCAACAAGCAAGGTA | AGTCGTACACATAGGTGGTCC |
| *Glut4* | GTGACTGGAACACTGGTCCTA | CCAGCCACGTTGCATTGTAG |
| *Cpt1a* | CTCCGCCTGAGCCATGAAG | CACCAGTGATGATGCCATTCT |
| *Acadm* | AGGGTTTAGTTTTGAGTTGACGG | CCCCGCTTTTGTCATATTCCG |
| *Acadvl* | CTACTGTGCTTCAGGGACAAC | CAAAGGACTTCGATTCTGCCC |
| *Acsl1* | TGCCAGAGCTGATTGACATTC | GGCATACCAGAAGGTGGTGAG |
